# Supplementary material for: Patterns of Intron Gain and Loss in Fungi
Source: PLoS Biol. 2004 Nov 30;2(12):e422. doi: 10.1371/journal.pbio.0020422 (PMC532390; doi:10.1371/journal.pbio.0020422)
Supplement: Table S1 — Also available at http://genes.mit.edu/NielsenEtAl/. (4.3 MB ZIP). [file pbio.0020422.st001.zip › NielsenEtAl/html/276.html]

AN5536.1.NCU04507.1.MG00101.1.FG04038.1


```
 CLUSTAL W (1.82) Multiple Sequence Alignments - Introns Inserted


Sequence 1: NCU04507.1	399 aa
Sequence 2: FG04038.1	388 aa
Sequence 3: MG00101.1	408 aa
Sequence 4: AN5536.1	396 aa
Alignment Length: 414 aa
Number Identitical Residues: 229 aa
Alignment Score (without introns) 9729


MG00101.1 	MASEPAAPSATSSNADKP-VAVVCVGMAG1SGKTTFMQRINAHLHGKKDPPYVINLDPAV
NCU04507.1	---MASTSASSAAKADQP-VAIVCVGMAG1SGKTTFMQQINAHLHGKKEPPYVINLDPAV
FG04038.1 	-MAESSTTAGEAPKASSPPVAIVCVGMAG1SGKTTFMRRINAHLHQNDTPPYVINLDPAV
AN5536.1  	-------------MANSP-VAVVCVGMAG1SGKTTFMQRINSHLHSKKQPPYVLNLDPAV
          	              *..* **:******* *******::**:*** :. ****:******

MG00101.1 	LNVPFDSNIDIRDSVNYKEVMKQYNLGPNGGILTSLNLFATKVDQILNLLEKRTARDTPE
NCU04507.1	THSPFESNIDIRDSVNYKEVMKQYNLGPNGGILTSLNLFATKVDQVLGLLEKRTAPK-PD
FG04038.1 	LSVPFESNIDIRDSVNYEEVMKQYNLGPNGGILTSLNLFATKVDQIVNLLEKRAQPD-PE
AN5536.1  	HTVPFESNIDIRDAINYKEVMKQYNLGPNGGILTSLNLYATKVDQIIALLEKRAAPN-PE
          	   **:*******::**:********************:******:: *****:  . *:

MG00101.1 	NAGRKPIEHILVDTPGQIEAFVWSASGTILLESLASSFPTVIAYVVDTPRTRSTSTFMSN
NCU04507.1	DPTHTPIKHILVDTPGQIEVFVWSASGQILLESLASSFPTVIAYIIDTPRTSSTSTFMSN
FG04038.1 	KPDRKPIDRILVDTPGQIEVFVWSASGTILLESLASSFPTVIAYVIDTPRTASTSTFMSN
AN5536.1  	NPAAKPIKHFLVDTPGQIEVFVWSASGSILLETLASSFPTVIAYIIDTPRTSSTSTFMSN
          	..  .**.::*********.******* ****:***********::***** ********

MG00101.1 	MLYACSILYKTKLPMILVFNKTDVQDASFAKEWMTDFDAFQAALREDEERNAFGSAEGES
NCU04507.1	MLYACSILYKTKLPMILVFNKSDVKDPAFAKEWMTDYDAFQAALQEDETNNAFGGAEG-S
FG04038.1 	MLYACSILYKTKLPMILVFNKTDVKDAEFAKEWMTDFEAFQEALRKDEESDELGGVEG-G
AN5536.1  	MLYACSILYKTKLPMILVFNKTDVQDAEFAKEWMTDFDAFQQALREEEESGQFGGEGGAG
          	*********************:**:*. ********::*** **:::*  . :*.  * .

MG00101.1 	GSG---GSGYMSSLLNSMSMMLDEFYAHLSVVGVSSMTGHGVDDFFTAVAEKAEEFRRDY
NCU04507.1	GDGMGSGSGYMGSLLNSMSLMLEEFYAHLNVVGVSSLYGTGIDEFFAAVQEKAEEFKRDY
FG04038.1 	GHG---GSGYMGSLLNSMSLMLEEFYAHLSVVGVSSRLGTGVDEFFEAVEEKRQEFLRDY
AN5536.1  	GMG---GSGYMGSLLNSMSLMLEEFYRHLNVVGVSAMTGEGVDEFFEAVEEKRKEFERDY
          	* *   *****.*******:**:*** **.*****:  * *:*:** ** ** :** ***

MG00101.1 	QPELERRREERDEAKKLIREQELDKMMKGMSFAGDKDGTGASGSNDAEDENPD-IAALGR
NCU04507.1	QPELERRREEREENKKKARERELNKMMKGMSMGDAAGDVTVGDVNDKEAP-----EPLST
FG04038.1 	QPELERKRAERDEQKKATREKELDKMMQGMSVGGLP-------VAKEEDD-----ADVAS
AN5536.1  	KPELERLKKEREEAQAKQREIELGKLMKDMNLSSSKAKEEPETVSEAEEEEDERVAAMGR
          	:***** : **:* :   ** **.*:*:.*....           . *  . .    :. 

MG00101.1 	RVDAIDSDDSVADDDEMDDEGLKARYDAALNDA-NASMSQDASFANYLRSQANQQ
NCU04507.1	DEESSDEEYGDDPNDEYDREGLQARYAAAMQGE-DDSVLADASFAKYLHSQR---
FG04038.1 	DDDGDD----TDSDEEIQKQGLQDRYEAAMGDQ-DDSVMADASFAKYLHTQR---
AN5536.1  	AYDPDSDSDEDYPPPAGDDEGLSQRYQSALADSRSGPSNEDASFMRYLQQSNLNG
          	  :  ...         : :**. ** :*: .  . .   **** .**: .  .
```
